# Concordance between the estimates of wasting measured by weight-for-height and by mid-upper arm circumference for classification of severity of nutrition crisis: analysis of population-representative surveys from humanitarian settings

**DOI:** 10.1186/s40795-018-0232-0

**Published:** 2018-05-18

**Authors:** Oleg Bilukha, Eva Leidman

**Affiliations:** Emergency Response and Recovery Branch, Division of Global Health Protection, Center for Global Health, Centers for Disease Control, 1600 Clifton Road, Atlanta, GA 30329 USA

**Keywords:** Wasting, Survey, Nutrition, Humanitarian

## Abstract

**Background:**

Despite frequent use of mid-upper arm circumference (MUAC) to assess populations at risk of nutrition emergencies, as well as evidence that measurement of children based on MUAC identifies different children than weight-for-height (WHZ) as wasted, no crisis classification thresholds based on prevalence of wasting by MUAC currently exist.

**Methods:**

We analyzed 733 population-representative anthropometric surveys from 41 countries conducted by Action Contre la Faim (ACF) and the United Nations High Commissioner for Refugees (UNHCR) between 2001 and 2016. Children aged 6–59 months were classified as wasted if they had a WHZ < − 2 and/or a MUAC < 125 mm. Prevalence of wasting as assessed by WHZ and by MUAC were compared using correlations and linear regression models adjusting for stunting prevalence, sex and age distribution of the sample. Median prevalence of wasting by MUAC corresponding to each of the WHZ-based crisis thresholds was examined.

**Results:**

Median prevalence of wasting by WHZ was 10.47% (IQR: 6.34–17.55%) and by MUAC was 6.66% (IQR:4.12–10.88%). Prevalence of wasting by WHZ exceeded prevalence by MUAC in 543 (74.1%) surveys and median prevalence by WHZ was greater in 30 (73.17%) countries. Prevalence of wasting by WHZ is poorly correlated with prevalence of wasting by MUAC (ρ = 0.55). R^2^ was 0.36 for unadjusted and 0.45 for adjusted linear regression model. The difference between the prevalence by WHZ and by MUAC increased as the overall prevalence by WHZ increased (ρ = 0.69). Surveys with prevalence of wasting by WHZ approximately equal to thresholds for “poor” (5% ± 2.5%), “serious” (10% ± 2.5%), “emergency” (15% ± 2.5%), and “famine” (30% ± 2.5%) were observed to have median prevalence of wasting by MUAC of 4.51% (IQR: 2.73–6.81%), 6.67% (IQR: 4.27–10.03%), 8.15% (IQR: 5.11–11.86%), and 15.71% (IQR: 10.28–17.50%), respectively. There was a very substantial overlap of MUAC values across the threshold categories.

**Conclusions:**

Given a poor correlation between population prevalence of wasting by WHZ and by MUAC, classification of surveys based on prevalence of wasting by MUAC will result in poor concordance with current WHZ-based crisis thresholds, even if regional differences are considered, regardless of the cutoffs used.

## Background

Prevalence of acute malnutrition is commonly used to benchmark the severity of a nutritional emergency to help inform the scale and scope of humanitarian response activities. Prevalence in a given context is compared with global standard thresholds. The World Health Organization (WHO) initially outlined guidance on these standard thresholds in 1995, modifying guidance from a 1992 consultation by the WHO Eastern Mediterranean regional office [1]. The guidance proposed classification of a situation using thresholds of less than 5% prevalence of wasting (“acceptable”), less than 10% (“poor”), less than 15% (“serious”) and equal to or greater than 15% (“critical”). The Management of Nutrition in Major Emergencies, a joint guidance document drafted in 2000 by WHO, United Nations High Commissioner for Refugees (UNHCR), International Federation of Red Cross (IFRC), and World Food Programme (WFP), included these same thresholds prompting a more universal adoption [2]. In 2004, the need for a higher, famine threshold was proposed by Howe and Devereux [3]. Currently, both the Integrated Phase Classification (IPC) used in East Africa and Asia and the Cadre Harmonisé (CH) used in the Sahel and West Africa use a cutoff of 30% prevalence of wasting as a threshold for famine, such that prevalence of wasting is used to classify a situation as Phase I (< 5%), Phase 2 (5- < 10%), Phase 3 (10- < 15%), Phase 4 (15- < 30%) or Phase 5 (≥ 30%) [4, 5].

The above standard thresholds are all based on prevalence of wasting as assessed by weight-for-height Z scores (WHZ) [1, 2, 4, 5]. In addition to WHZ, wasting can be assessed using mid-upper arm circumference (MUAC). Since in 2005 WHO, WFP, United Nations Children’s Fund (UNICEF), and the Standing Committee on Nutrition (SCN) recommended MUAC as independent measure of wasting used as a criterion for admission into selective nutrition feeding programs [6–8]. However, separate thresholds for classifying a crisis based on prevalence of wasting as assessed by MUAC do not exist. Previous research demonstrating substantial discrepancy in diagnosis of children as wasted using WHZ and MUAC has prompted questions about the validity of applying WHZ-based thresholds to estimates of wasting based on MUAC. Based on an analysis of over 560 surveys from 31 countries, WHO estimated that only about 4 in 10 children were identified as wasted by both WHZ and MUAC, concluding that “the cases selected using weight-for-height and MUAC were not the same” [6]. Multi-country analysis by Grellety et al. using 1832 surveys from 47 countries similarly highlighted that a large proportion of children were identified as wasted by MUAC but not WHZ and by WHZ but not MUAC, adding that the proportion of children in each of these categories varied widely by country [9]. Analysis by Roberfroid et al. found that stunting, sex, and age all influenced diagnosis of acute malnutrition by MUAC but not WHZ [10]. However, while there is evidence to suggest poor correlation between WHZ and MUAC diagnosis of individual children, whether or not this translates into population level differences in prevalence of wasting by WHZ and MUAC has yet to be evaluated.

Mid-upper arm circumference is increasingly used to measure wasting, especially as part of community-based screenings and at remote clinics where height boards and other anthropometric equipment may not be available. Additionally, several studies suggest that low MUAC better predicts mortality than low WHZ, as summarized by Briend et al [11]. MUAC only assessments are particularly common in humanitarian settings with extreme insecurity. In the absence of clear guidance on thresholds for classifying prevalence of acute malnutrition by MUAC, the WHZ-based thresholds have been applied in many contexts. The Cadre Harmonisé, for example, recommends this approach for the Sahel and West Africa [4]. The Integrated Food Security and Nutrition Phase Classification technical committee has identified the need for secondary analysis of existing survey data to explore the possibility of deriving the thresholds for classifying severity of wasting at the population level using prevalence of wasting by MUAC where WHZ based anthropometry data are not available.

The objective of this research therefore was to explore the concordance of the prevalence of wasting by WHZ and MUAC at the population level and the possibility of deriving MUAC-based crisis thresholds corresponding to the existing WHZ-based thresholds. The focus of the analysis was on total rather than severe wasting, as WHO-recommended emergency thresholds are based on the prevalence of total wasting [1, 2]. To this aim, we assessed the correlation of prevalence of low MUAC and low WHZ in survey samples globally, as well as described prevalence of wasting by MUAC in populations with prevalence approximately equal to poor, serious, critical, and famine thresholds as determined by WHZ.

## Methods

Data included in these analyses were from small-scale field nutrition surveys conducted in humanitarian settings by Action Contre la Faim (ACF) International (an international humanitarian non-governmental organization focused on nutrition in humanitarian settings worldwide) and by the United Nations High Commissioner for Refugees (UNHCR). Data were drawn from a database of 808 population-representative cross sectional surveys conducted between 2001 and 2016 [12, 13]. Surveys with sample sizes smaller than 196 persons and cluster surveys with fewer than 25 clusters were excluded a priori from all analyses as they did not meet minimum standards for small-scale cluster surveys [9]. Surveys that did not collect both MUAC and weight-for-height (weight, height, age and sex) were also excluded.

Weight-for-Height Z-scores (WHZ) were calculated for each child using the WHO 2006 growth standards using the WHO SAS macro [14]. Only children aged 6–59 months were included in the analyses. Prevalence of wasting by WHZ for each survey reflects the proportion of children with WHZ less than − 2. Outlier observations were excluded from a survey if Z-score of a child fell outside the flexible exclusion range of ±4 Z-scores from the observed survey sample mean, as described by WHO [1]. Prevalence of wasting by MUAC for each survey reflects the proportion of children with MUAC values less than 125 mm. MUAC values less than 70 mm and greater than 220 mm were excluded as outliers. Individual observations within each survey were also excluded from calculations of wasting by WHZ for children without information on height, weight, age or sex and from calculations of wasting by MUAC for children without information on MUAC and age. Cases of bilateral pitting edema were not included in estimated prevalence of wasting by WHZ or MUAC; edema cases were relatively rare in all surveys, representing approximately 3 per 1000 (mean: 0.32%) sampled children.

Countries where the surveys were conducted were categorized into seven geographical or country groupings (Latin America and the Caribbean; Eastern and Southern Africa; Democratic Republic of Congo (DRC); West and Central Africa; South, Southeast Asia and Pacific; Sudan; Middle East and North Africa) as seen in Table 1. DRC and Sudan were analyzed as its own grouping given the large number of surveys conducted in both countries.

**Table 1:** Number of included surveys, children and prevalence of wasting by country and region, 2001–2016

	Number of surveys	Number of children	Prevalence of wasting by WHZ Median (IQR)	Prevalence of wasting by MUAC Median (IQR)
Latin America and the Caribbean	16	11,808	3.95% (2.87–4.46)	2.99% (2.17–4.35)
Bolivia	1	884	1.28%	0.35%
Guatemala	2	1432	2.10% (0.39–3.80)	3.83% (0.20–7.46)
Haiti	13	9492	4.27% (3.81–4.55)	3.00% (2.77–4.15)
Eastern and Southern Africa	190	128,404	9.15% (5.76–17.88)	4.66% (2.91–7.49)
Angola	1	870	5.77%	4.53%
Botswana	1	215	3.66%	0.61%
Burundi	7	3493	5.76% (4.23–7.08)	3.03% (2.08–9.33)
Eritrea	2	859	20.22% (18.89–21.55)	4.31% (4.02–4.60)
Ethiopia	59	29,554	16.93% (8.94–21.77)	4.95% (3.59–8.50)
Kenya	46	30,173	10.92% (7.88–15.73)	3.74% (2.66–5.28)
Madagascar	1	635	7.47%	6.45%
Mozambique	1	406	3.26%	3.26%
Rwanda	13	4694	4.74% (3.85–5.97)	1.98% (1.82–3.11)
Somalia	3	2688	15.85% (15.10–37.79)	5.70% (2.22–25.35)
South Sudan	17	15,518	11.4% (8.19–22.15)	7.55% (6.17–9.84)
Tanzania	5	2487	1.90% (1.54–2.61)	1.18% (0.74–3.33)
Uganda	32	36,022	4.93% (3.76–8.92)	6.78% (4.34–9.08)
Zambia	2	790	4.40% (4.38–4.42)	6.38% (2.35–10.40)
Democratic Republic of Congo	130	118,871	7.40% (4.36–10.46)	7.97% (5.36–12.51)
West and Central Africa	146	90,857	9.60% (6.31–14.99)	5.89% (3.00–9.69)
Burkina Faso	11	5593	12.41% (6.21–14.99)	6.11% (2.51–8.78)
Cameroon	12	5614	9.24% (7.33–12.28)	6.86% (4.65–7.92)
Central African Republic	8	6357	5.75% (5.40–6.63)	8.02% (6.78–9.04)
Chad	67	39,428	9.66% (6.34–18.89)	3.76% (2.06–11.09)
Guinea	5	4054	6.01% (5.16–7.81)	3.67% (2.88–4.80)
Liberia	9	4518	3.57% (3.13–4.39)	4.00% (1.98–5.43)
Mali	8	6342	10.74% (9.20–16.41)	6.29% (5.71–7.33)
Mauritania	6	3693	12.61% (8.77–14.81)	5.36% (4.14–7.90)
Niger	14	9825	13.08% (11.07–15.95)	8.58% (6.53–13.32)
Sierra Leone	6	5433	7.01% (6.15–7.13)	9.89% (5.88–10.67)
South East Asia and Pacific	87	63,411	11.95% (7.36–17.20)	8.00% (4.54–13.28)
Afghanistan	24	21,220	8.57% (5.90–11.41)	9.06% (6.46–14.38)
Bangladesh	22	11,373	12.69% (9.70–14.25)	5.35% (4.28–6.74)
India	1	465	20.58%	10.07%
Myanmar	13	9993	18.26% (5.48–20.75)	11.64% (9.66–15.05)
Nepal	9	5946	14.31% (12.50–19.58)	10.55% (6.49–15.32)
Pakistan	13	10,887	18.45% (11.40–19.55)	9.76% (6.92–14.79)
Philippines	4	2630	6.06% (4.77–8.64)	1.21% (0.88–1.42)
Tajikistan	1	897	9.81%	16.01%
Sudan	150	130,735	18.96% (14.06–23.82)	10.21% (7.05–14.26)
Middle East North Africa	14	5602	6.69% (2.28–12.02)	3.46% (1.23–5.75)
Djibouti	6	2190	11.42% (10.39–13.84)	5.20% (3.78–7.27)
Iraq	1	575	2.28%	3.14%
Jordan	4	1610	1.18% (0.88–2.04)	1.01% (0.69–1.15)
Yemen	3	1227	5.59% (3.61–17.17)	4.14% (2.38–6.53)
All Surveys	733	549,688	10.47% (6.34–17.55)	6.66% (4.12–10.88)

The first aim of the analysis was to describe the relationship between prevalence of wasting as assessed by WHZ and the prevalence of wasting as assessed by MUAC on the same population. Spearman correlations were therefore calculated to describe the correlation between prevalence of wasting by WHZ and by MUAC, as well as between the difference in prevalence by WHZ and MUAC and the prevalence by each WHZ and MUAC survey. A multivariate model was then constructed to explore the relationship of the prevalence by WHZ and by MUAC, controlling for key factors shown in previous research to be associated with the prevalence of wasting by MUAC. These factors included stunting prevalence, sex distribution, and age distribution of the survey sample [10]. Prevalence of wasting by MUAC as an outcome and all predictor variables were modeled as continuous linear terms. Sex ratio was calculated as the proportion of females in the survey sample. Age ratio was calculated as the proportion of younger children aged 6–29 months in the survey sample. Observations with significantly high leverage or Cook’s distance were removed from the multivariable analyses. The regression analysis was repeated using logit-transformed independent and dependent variables [15]. To assess the reproducibility of the results, analysis above was repeated with DRC included in the West and Central Africa region and Sudan included in the Middle East North Africa, and separately for surveys conducted by ACF and UNHCR.

Second, we described the prevalence of wasting by MUAC in surveys with the prevalence of wasting by WHZ corresponding to the existing WHZ-based crisis thresholds (5, 10, 15 and 30%) to assess the feasibility of deriving corresponding thresholds using MUAC. Surveys with prevalence of wasting by WHZ within ±2.5% of the 5, 10, 15, and 30% crisis thresholds were included in the analysis. For example, to explore prevalence of MUAC that may correspond to the 10% WHZ-based crisis threshold we used surveys with a prevalence of wasting by WHZ between 7.5 and 12.5%. Median and interquartile range (IQR) for prevalence of wasting by MUAC were calculated for each of the sub-sets of surveys with prevalence approximately equal to the four thresholds, overall and by geographic region. The analysis was repeated using only the surveys within ±1.5% of the crisis thresholds.

Finally, we explored concordance of the possible MUAC classification by determining the proportion of surveys that would be classified into the same crisis category if categorized separately based on prevalence of wasting by WHZ and by MUAC. An example set of MUAC thresholds was used for this classification, derived from the observed median values observed in the previous stage analysis.

All data were aggregated and cleaned using SAS Version 9.3, analysis was performed in Stata IC Version 14.2, and figures were produced in JMP Version 13.0.0.

## Results

In total, 808 surveys were reviewed for this study. Seventy-five surveys were excluded from the analysis: 60 surveys did not collect MUAC measurements and another 15 had fewer than 25 clusters and/or had a sample size smaller than 196 children, resulting in 733 surveys from 41 countries retained for analysis. As seen in Table 1, the countries with the largest number of surveys were Sudan (150 surveys), DRC (130), Chad (67), Ethiopia (59) and Kenya (46). All other countries had 32 of fewer surveys each. Among selected surveys, 0.7% of children aged 6–59 months were excluded due to missing anthropometric values (sex, weight, height or MUAC) and an additional 0.4% were excluded due to out of range values for WHZ or MUAC. After exclusions, these surveys represent data from approximately 550 thousand children.

Prevalence of wasting by WHZ was higher than by MUAC for most surveys. Median prevalence of wasting by WHZ was 10.47% (IRQ: 6.34–17.55%) and by MUAC was 6.66% (IQR: 4.12–10.88%) (Table 1). Prevalence of wasting by WHZ exceeded prevalence by MUAC in 543 (74.1%) surveys and median prevalence by WHZ was greater in 30 (73.17%) countries. The difference in median prevalence was greatest among surveys in East Africa such as Eritrea (15.91%), Ethiopia (11.98%), and Somalia (10.15%) as well as in India (10.51%).

**Fig. 1:**
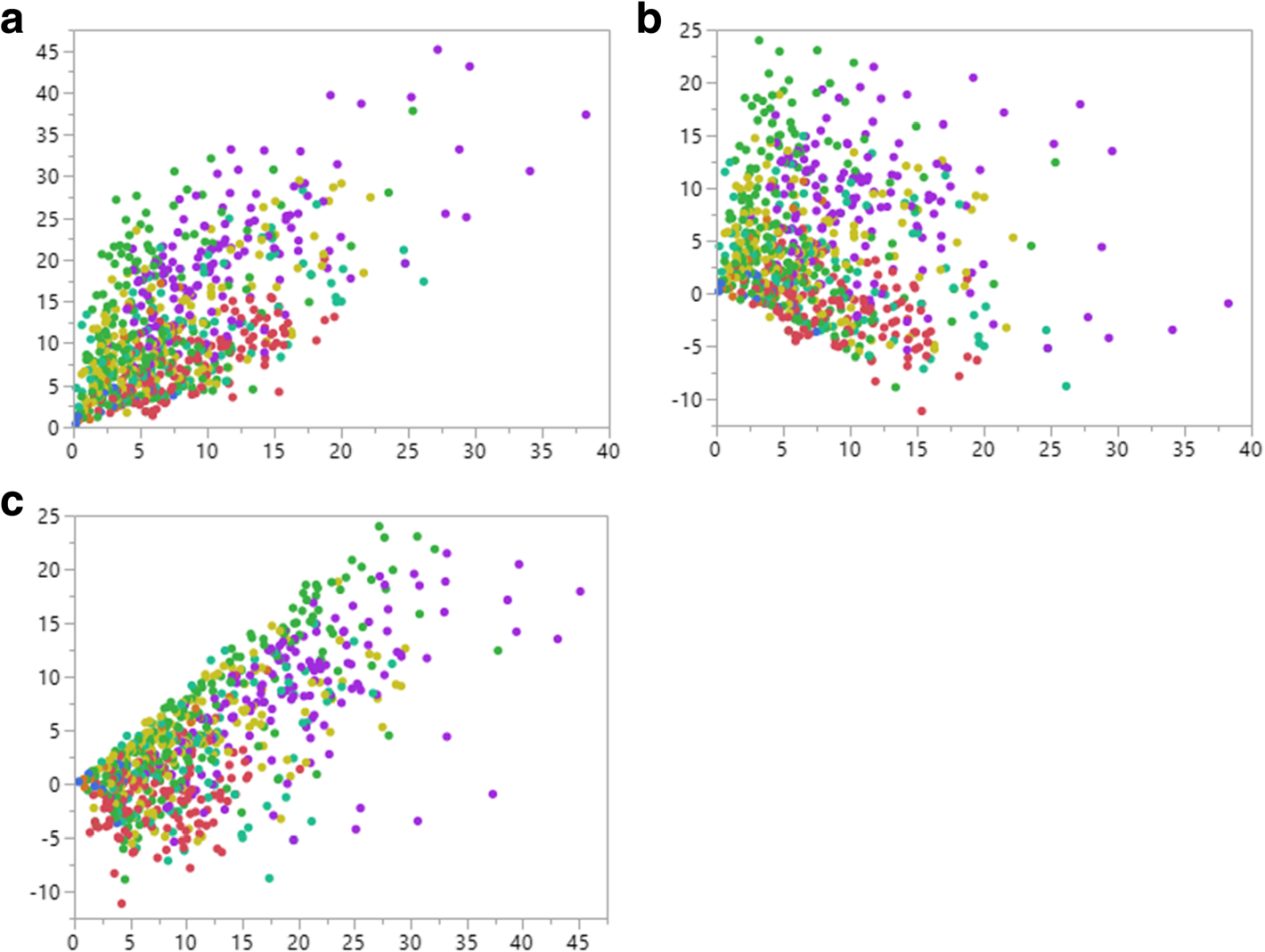
Correlation of prevalence of wasting by weight-for-height, mid-upper arm circumference, and difference in prevalence. **a** Correlation of prevalence of global acute malnutrition (GAM) as determined by weight-for-height (Y-axis) and prevalence of GAM by mid-upper arm circumference (MUAC) (X-axis), by region. **b** Correlation of the difference in prevalence of GAM as determined by WHZ and prevalence of GAM by MUAC (Y-axis) and prevalence of GAM by MUAC (X-axis), by region. **c** Correlation of the difference in prevalence of GAM as determined by WHZ and prevalence of GAM by MUAC (Y-axis) and prevalence of GAM by WHZ (X-axis), by region. Regions represented by colors as follows: Latin America and the Caribbean (blue), Eastern and Southern Africa (green), Congo DRC (red), West and Central Africa (yellow), Middle East and North Africa (orange), South East Asia and Pacific (aqua), Sudan (purple)

The data suggest a positive but relatively weak monotonic correlation (ρ = 0.5485) between prevalence of wasting by WHZ and by MUAC (Table 2 and Fig. 1a). By region, correlation was highest for the Middle East and North Africa (ρ = 0.8901) and DRC (ρ = 0.6822) and lowest for Eastern and Southern Africa (ρ = 0.3553). Rho for all surveys was improved when prevalence of wasting by WHZ was correlated with the difference between the prevalence of wasting by WHZ and by MUAC (ρ = 0.6859). Notably, difference in prevalence by WHZ and by MUAC was greatest for surveys with higher prevalence of wasting by WHZ. Conversely, overall correlation was lowest when prevalence of wasting by MUAC was correlated with the difference between the prevalence of wasting by WHZ and by MUAC (ρ = − 0.1634). The strength of the correlation varied by region (Table 2 and Fig. 1b, c). These correlations did not change markedly when surveys from ACF and UNHCR were analyzed separately (not presented).

**Table 2:** Spearman’s correlation (rho) for prevalence of wasting by weight-for-height and by mid-upper arm circumference overall and differences in prevalence^a^

	WHZ v. MUAC	(WHZ- MUAC) v MUAC	(WHZ-MUAC) v WHZ
Latin America and Caribbean	0.3667	−0.6377	0.3726
Eastern and Southern Africa	0.3553	−0.1599	**0.8279**
Congo, DRC	**0.6822**	−0.4811	0.2513
West and Central Africa	**0.6247**	−0.0886	**0.6652**
Sudan	**0.6187**	−0.1317	**0.6210**
Middle East North Africa	**0.8901**	**0.6352**	**0.8857**
South East Asia and Pacific	**0.5639**	−0.4296	0.4501
All Surveys	**0.5485**	−0.1634	**0.6859**

^a^Rho values greater than 0.5 are indicated in bold

R^2^ in the univariate linear model with prevalence of WHZ as a predictor and prevalence by MUAC as an outcome was 0.36. R^2^ in the multivariate model adjusted for prevalence of stunting, the proportion of younger children (aged 6–29 months of age), and the proportion of females, increased to 0.46 (Table 3). Multivariate model results suggest that a 1% increase in prevalence of wasting by WHZ was associated with a 0.5% increase in prevalence of wasting by MUAC; this association was highly significant (*p* < 0.001). All other co-variates were also positively associated with prevalence of wasting by MUAC. Prevalence of stunting and the proportion of younger children were both significant (*p* < 0.001 for both), whereas the proportion of females was not (*p* = 0.218) (Table 3). Logit transformation of all variables in the model did not improve fit of either univariate or multivariate models (R^2^ = 0.35 and 0.43, respectively).

**Table 3:** Parameter estimates from univariate and multivariate linear regression models of prevalence of wasting as assessed by mid-upper arm circumference

	Univariate		Multivariate	
Dependent variables	Coefficient	95% CI	Coefficient	95% CI
Intercept	2.74	(2.14–3.34)^††^	−9.36	(−15.03–3.70)^†^
Prevalence of Wasting, Weight-for-Height < −2Z	0.43	(0.39–0.47)^††^	0.50	(0.46–0.54)^††^
Prevalence of Stunting, Height-for-Age < −2Z			0.11	(0.09–0.12)^††^
Percent of Children less than 30 Months of Age			0.08	(0.03–0.14)^††^
Percent Females			0.06	(−0.04–0.17)
Model R^2^	0.3623		0.4560	

^†^Statistically significant, *p* < 0.05

^††^Statistically significant, *p* < 0.001

**Fig. 2:**
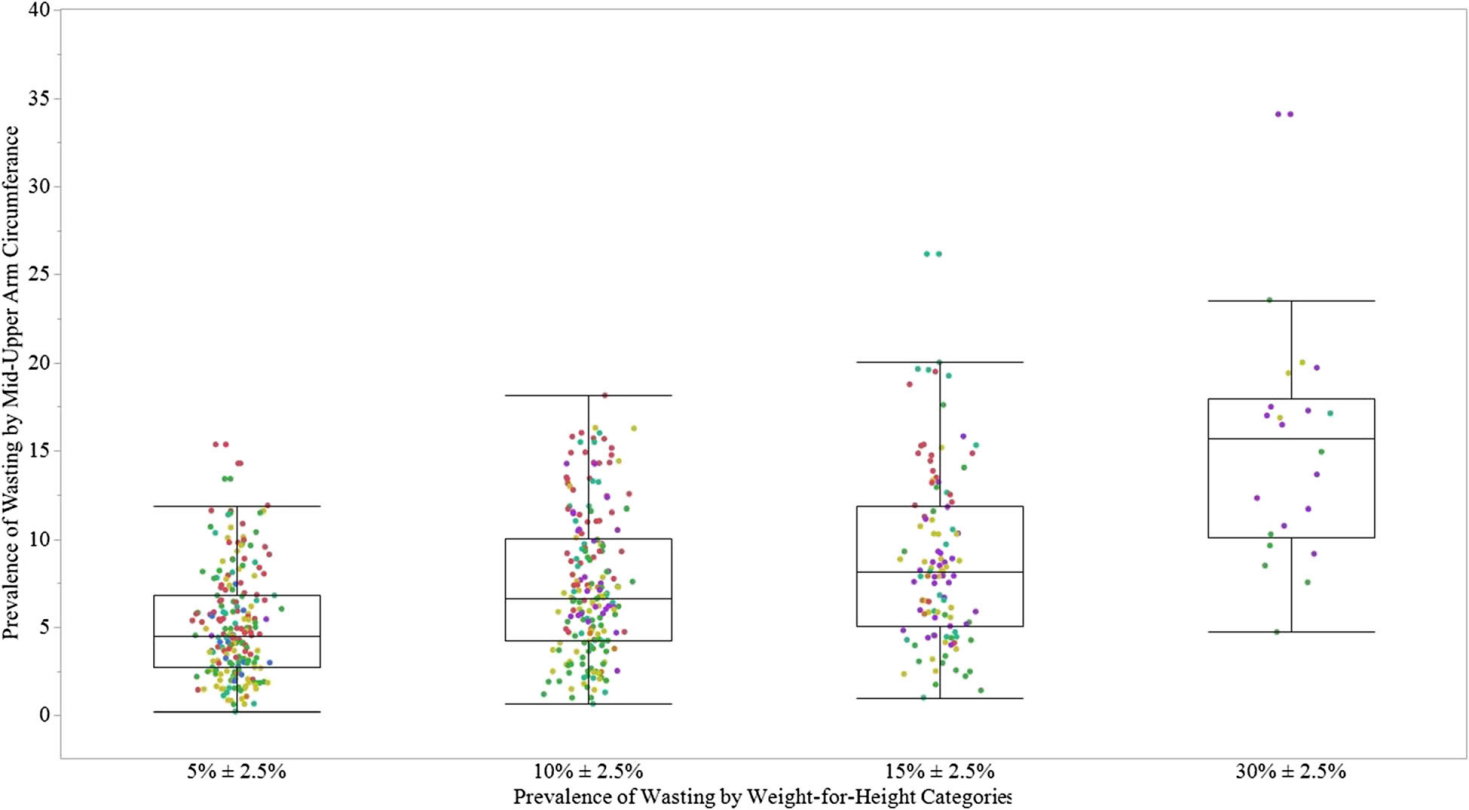
Box-plots for prevalence of wasting by mid-upper arm circumference for surveys corresponding to existing crisis classification thresholds. Regions represented by colors as follows: Latin America and the Caribbean (blue), Eastern and Southern Africa (green), Congo DRC (red), West and Central Africa (yellow), Middle East and North Africa (orange), South East Asia and Pacific (aqua), Sudan (purple)

Table 4 and Fig. 2 present the median prevalence of wasting by MUAC corresponding to each of the WHZ-based crisis thresholds (5, 10, 15, and 30%). Overall, median prevalence of wasting by MUAC was 4.51% (IQR: 2.73–6.81%) for surveys near the “poor” threshold (5 ± 2.5%), 6.67% (IQR: 4.27–10.03%) for surveys near the “serious” threshold (10 ± 2.5%), 8.15% (IQR: 5.11–11.86%) for surveys near the “emergency” threshold (15 ± 2.5%), and 15.71% (IQR: 10.28–17.50%) for surveys near the “famine” threshold (30 ± 2.5%). Median MUAC thresholds corresponding to 5, 10, 15% were virtually unchanged when only surveys within ±1.5% of the thresholds rather than ±2.5% of the thresholds were included in the analysis: 4.46, 7.06, and 7.92%, respectively. However, median wasting prevalence by MUAC corresponding to the famine threshold (30%) was higher when only surveys within ±1.5% of the thresholds were included (16.94% vs. 15.71%); this estimate is likely less stable due to the smaller number of surveys in this threshold category.

**Table 4:** Median and interquartile range for prevalence of wasting by MUAC for surveys corresponding to existing nutritional crisis classification thresholds, by region

	Prevalence of wasting by MUAC, [N] Median (IQR)			
	Poor	Serious	Emergency	Famine
	5% ± 2.5%	10% ± 2.5%	15% ± 2.5%	30% ± 2.5%
Eastern and Southern Africa	[61] 4.10% (2.49–6.45)	[56] 4.41% (2.89–6.96)	[18] 4.12% (2.55–9.30)	[7] 9.64% (7.55–14.95)
Congo, DRC	[60] 5.66% (4.46–7.99)	[46] 11.04% (7.75–14.30)	[17] 13.86% (12.08–14.86)	
West and Central Africa	[52] 3.35% (1.85–5.61)	[42] 6.02% (3.61–7.22)	[26] 8.29% (5.86–10.28)	[3] 19.41% (16.88–20.02)
Sudan	[2] 4.98% (4.51–5.46)	[27] 7.49% (5.78–10.52)	[28] 7.89% (5.72–9.05)	[11] 16.48% (11.70–17.50)
South East Asia and Pacific	[21] 5.21% (2.73–6.81)	[25] 7.03% (4.64–11.85)	[20] 8.03% (4.63–17.29)	[1] 17.13%
All Surveys^a^	[211] 4.51% (2.73–6.81)	[200] 6.67% (4.27–10.03)	[112] 8.15% (5.11–11.86)	[22] 15.71% (10.28–17.50)
	5% ± 1.5%	10% ± 1.5%	15% ± 1.5%	30% ± 1.5%
Eastern and Southern Africa	[40] 4.04% (2.47–7.29)	[31] 5.03% (2.50–7.59)	[12] 3.62% (2.35–9.31)	[2] 11.25% (7.55–14.95)
Congo, DRC	[37] 5.85% (4.91–8.86)	[32] 11.22% (8.25–13.88)	[11] 13.49% (11.9–14.86)	
West and Central Africa	[32] 3.53% (1.91–5.61)	[25] 6.20% (3.71–7.22)	[15] 8.12% (5.56–8.78)	[3] 19.41% (16.88–20.02)
Sudan	[1] 5.46%	[17] 7.11% (5.78–9.91)	[15] 7.92% (5.17–8.90)	[7] 17.00% (12.32–19.72)
South East Asia and Pacific	[15] 5.21% (1.55–7.81)	[15] 7.03% (4.64–11.04)	[10] 6.23% (4.46–19.60)	
All Surveys^a^	[183] 4.46% (2.58–7.25)	[122] 7.06% (4.64–10.98)	[64] 7.92% (4.46–11.99)	[12] 16.94% (13.63–19.57)

^a^Surveys from Latin America and Caribbean region and the Middle East North Africa region are included among “All Surveys” but not presented separately by region given small numbers of surveys per category

Prevalence of wasting by MUAC for surveys with WHZ prevalence near all four thresholds varied considerably, as illustrated by the wide interquartile ranges and overall distributions (Fig. 2). For example, for surveys with wasting prevalence by WHZ approximately equal to 10%, prevalence of wasting by MUAC ranged from less than 1% to nearly 20%. The distributions for each of the four threshold categories all overlap substantially. Nearly half (48.0%) of all surveys corresponding to the 10% threshold (± 2.5%), have prevalence of wasting within the IQR for the 15% threshold (± 2.5%), too great an overlap to allow for meaningful discriminatory power.

Median prevalence of wasting by MUAC for each threshold category varied by region, suggesting that regional variation contributed to the overall variability observed. For surveys with prevalence of wasting by WHZ approximately equal to the 5, 10 and 15% thresholds, median wasting prevalence by MUAC was greatest in the DRC. Median wasting by MUAC in DRC was nearly double that of surveys from West and Central Africa for surveys with wasting by WHZ of 5 ± 2.5 and 10% ±2.5% and more than triple that of surveys from Eastern and Southern Africa with wasting prevalence by WHZ of 15 ± 2.5%. Median prevalence of wasting by MUAC was also lowest in Eastern and Southern Africa for surveys near the famine threshold (30 ± 2.5%).

Due to the large observed variation in wasting prevalence by MUAC corresponding to the current WHZ-based crisis thresholds, classification of surveys based on prevalence of MUAC and WHZ independently resulted in poor concordance regardless of the MUAC-based thresholds used. Table 5 presents as illustration the proportion of surveys that would be classified into the same crisis category using a dozen MUAC threshold combinations derived based on analysis presented in Table 4 when compared with WHZ-categories of 5, 15 and 30%. In all iterations, approximately 4 in 10 surveys were classified into the same crisis category. No combination of MUAC-based thresholds achieved greater than 50% concordance. Notably, Table 5 only contains suggested MUAC thresholds corresponding to 5, 15 and 30% WHZ thresholds. As shown in the previous analyses, the overlap in MUAC values around 10 and 15% WHZ thresholds was too great to suggest a separate meaningful MUAC threshold for 10% WHZ threshold. Including this threshold generally resulted in a lower proportion of concordant surveys.

**Table 5:** Classification into crisis categories surveys by prevalence of wasting by mid-upper arm circumference

	Crisis categories based on prevalence of wasting by mid-upper arm circumference				Percent concordance (*N* = 733)
	0- < 5% Acceptable	5- < 15% Poor/Serious	15- < 30% Emergency	≥ 30% Famine	
1	< 4%	4- < 7%	7- < 16%	≥ 16%	41.88%
2	< 4%	4- < 7%	7- < 17%	≥ 17%	42.84%
3	< 4%	4- < 8%	8- < 16%	≥ 16%	44.75%
4	< 4%	4- < 8%	8- < 17%	≥ 17%	45.70%
5	< 4%	4- < 9%	9- < 16%	≥ 16%	46.38%
6	< 4%	4- < 9%	9- < 17%	≥ 17%	47.34%
7	< 5%	5- < 7%	7- < 16%	≥ 16%	37.93%
8	< 5%	5- < 7%	7- < 17%	≥ 17%	38.88%
9	< 5%	5- < 8%	8- < 16%	≥ 16%	40.79%
10	< 5%	5- < 8%	8- < 17%	≥ 17%	41.75%
11	< 5%	5- < 9%	9- < 16%	≥ 16%	42.43%
12	< 5%	5- < 9%	9- < 17%	≥ 17%	43.38%

## Discussion

Analysis presented in this paper aimed to assess the feasibility of developing thresholds for determining the severity of a crisis in contexts where assessments of wasting using weight-for-height, the indicator for which WHO recommended emergency thresholds exist, were not practical and only mid-upper arm circumference could be measured. However, analysis of survey data from over 700 surveys from more than 40 countries suggests that prevalence of wasting as assessed by MUAC was poorly correlated with prevalence of wasting by WHZ. Correlation was not substantively improved when analysis was repeated separately by region; rho values for all regions were below 0.7. Consistent with previous literature [10], multivariable model demonstrated that an increase in prevalence of wasting by MUAC was significantly associated with an increased prevalence of stunting and an increased proportion of younger children (6 to 29 months of age) in the survey sample. Proportion of females in the sample was not significantly associated with the prevalence of wasting by MUAC. However, while prevalence of stunting and the proportion of younger children were both significant, including them in the model did not markedly improve fit (R^2^ multivariate = 0.46; R^2^ univariate = 0.36). A poor correlation of wasting prevalence as assessed by WHZ and MUAC is consistent with previous literature on inconsistencies in diagnosis of individual children as wasted using WHZ and MUAC [6, 9, 10].

Prevalence of wasting in most contexts was higher when assessed by WHZ than MUAC, however the reverse was true in approximately a quarter of all surveys. The difference in prevalence of wasting by WHZ and MUAC varied considerably, even within the same country and region. Interestingly, our analysis suggest that this difference in prevalence was more strongly correlated with prevalence of wasting by WHZ (ρ = 0.69) than the correlation of the two prevalence estimates directly (ρ = 0.55). On the other hand, this difference in prevalence was poorly correlated with the prevalence of wasting by MUAC (ρ = − 0.16). Given the main focus of this analysis on predicting prevalence of wasting by WHZ in contexts where only wasting by MUAC is known, the high correlation of wasting by WHZ and the difference in wasting prevalence has limited practical utility; wasting by MUAC has very little predictive power on the difference between the prevalence by WHZ and MUAC.

Developing an algorithm for conversion between prevalence of wasting by MUAC and WHZ, or converting WHZ-based thresholds to MUAC-based thresholds, is inadvisable given the observed correlation and high heteroscedasticity. Previous research deriving formulas for converting between prevalence estimates have been based on much stronger correlations. For example, the algorithm to convert from estimates of child malnutrition using the National Center for Health Statistics (NCHS) growth reference to estimates using the new WHO growth standards was based on a very high degree of fit (R^2^ > 0.9) of the regression models used to derive conversion formulas; R^2^ for wasting model was 0.96 [15].

The follow-up analysis presented in Tables 4 and 5 and Fig. 2 illustrates how poor correlation between prevalence of wasting by WHZ and MUAC translates into frequent discordant classification using MUAC- and WHZ-based thresholds. For surveys with prevalence of wasting by WHZ within ±1.5% of each of the crisis thresholds, the prevalence of wasting by MUAC varied by 15% points or more for each threshold. The box plots for wasting prevalence by MUAC for surveys near thresholds of 10 and 15% as assessed by WHZ overlapped almost entirely. Even when serious and emergency categories were combined, no combination of thresholds resulted in concordant phase classification of wasting by WHZ and MUAC for more than half of the surveys. Further, the data do not support the development of region-specific thresholds. While median prevalence of wasting by MUAC for surveys within ±1.5% of each of the crisis thresholds did vary by region, within regions the variability of prevalence around the median remained high. Findings on poor correlation between prevalence of wasting by WHZ and MUAC confirm that MUAC-based thresholds cannot be derived from WHZ-based thresholds. They also highlight the broader issue of the lack of evidence that underlies the indicators currently used to classify severity of emergencies. Further research would be needed to develop MUAC-based thresholds that are independent of the current WHZ-based thresholds and objectively define crisis severity, potentially considering functional outcomes (e.g., morbidity, mortality), and response to treatment.

This analysis is subject to several limitations. First, included surveys disproportionately represent countries with refugees, displaced persons, and/or experiencing chronic nutrition emergencies—Democratic Republic of Congo, Sudan, Chad, Ethiopia, Kenya. Surveys were all conducted by one of two agencies (ACF and UNHCR). This analysis includes only surveys made available for analysis. If repeated on a different set of surveys, this analysis may yield slightly different estimates, however the overall pattern of relationship is not likely to change. Second, the dataset contains relatively few surveys with very high prevalence (near famine thresholds) reflecting the fact that these contexts are fortunately relatively rare. Estimates of wasting prevalence by MUAC corresponding with high wasting prevalence by WHZ are therefore less stable compared with estimates for lower prevalence thresholds. Finally, while available data allowed for adjustment in regression analysis for key predictors of MUAC such as stunting, age and sex distribution, data on sitting-to-standing height ratio, another moderately important predictor of wasting by MUAC diagnosis was not measured in the surveys [10].

## Conclusion

In summary, estimates of wasting from MUAC-only assessments, such as those collected in humanitarian contexts with great insecurity, cannot be reliably converted to estimates of wasting by WHZ as prevalence of wasting assessed by MUAC is poorly correlated with prevalence wasting assessed by WHZ. As such, data presented in this analysis does not support the development of MUAC-based crisis thresholds corresponding with the current WHZ-based thresholds. In addition, applying current WHZ-based thresholds (5, 10, 15, 30%) to prevalence of wasting assessed by MUAC is not recommended, especially in contexts with high prevalence of wasting by WHZ. As demonstrated, the difference in prevalence of wasting as assessed by WHZ and by MUAC is greatest in crises when wasting becomes more prevalent, so the estimates from MUAC-only assessments will tend to provide lower estimates of crisis severity than WHZ.

## Abbreviations

ACF: Action Contre la Faim
CH: Cadre Harmonisé
DRC: Democratic Republic of Congo
IFRC: International Federation of Red Cross
IPC: Integrated Phase Classification
IQR: Interquartile range
MUAC: Mid-upper arm circumference
SCN: Standing Committee on Nutrition
UNHCR: United Nations High Commissioner for Refugees
UNICEF: United Nations Children’s Fund
WHO: World Health Organization
WHZ: Weight-for-height Z scores
